# A case report of thyroid myofibroblastic sarcoma

**DOI:** 10.1097/MD.0000000000043992

**Published:** 2025-08-29

**Authors:** Liuxue Ma, Huijuan Wang, Xin Li, Zixin Dai, Jianwei Zhang, Jinxia Gao

**Affiliations:** a Liangzhou Hospital, Wuwei, China; b Gansu Cancer Hospital, Lanzhou, China.

**Keywords:** clinical features, myofibroblastic sarcoma, thyroid

## Abstract

**Rationale:**

High-grade myofibroblastic sarcoma (HGMS) is a rare mesenchymal tumor with a high recurrence and metastatic rate. Few cases of high-grade myofibroblastic sarcomas have been reported. Herein, we report the first case of HGMS originating from the thyroid.

**Diagnosis and patient concerns:**

Based on the findings of thyroid, pathological examination, and immunohistochemical staining, grade III myofibroblastic sarcoma (MS) was diagnosed. Meanwhile, systemic imaging revealed multiple metastases in the scalp, lung, bone, liver, spleen, pancreas.

**Interventions and outcome:**

The patient had not received any treatment and expired 28 days after admission.

**Lessons:**

HGMS of thyroid shows high invasiveness and metastatic potential, often leading to rapid disease progression and poor survival outcomes. Early diagnosis and intervention guided by clinical evaluation, pathological findings, and imaging are essential to improve patient prognosis.

## 1. Introduction

Myofibroblastic tumors are uncommon mesenchymal neoplasms characterized by the proliferation of myofibroblasts, demonstrating features of both fibroblasts and smooth muscle cells. Malignant myofibroblastic sarcoma (MFS) represents a rare subtype of soft tissue sarcoma. Due to its low incidence, epidemiological data on MFS are limited and largely derived from large-scale cancer registries or multinational collaborative studies. Based on biological behavior, these tumors are categorized into 3 groups: benign, intermediate, and malignant. Malignant MFS accounts for approximately 20% of all cases. Its malignancy is considered to fall between that of low-grade sarcomas and more aggressive invasive tumors.^[1,2]^ MFS is a rare spindle cell malignancy that primarily occurs in the head and neck region and can affect adults and children.^[3,4]^ Most research on MFS consists of individual case reports, with no comprehensive epidemiological studies currently available.^[5]^ MFS displays a spectrum of histopathological features ranging from low- to high-grade, with distinct subtypes presenting variable clinical manifestations and prognoses.

However, all forms share a common basis in diagnosing myofibroblastic tumor.^[6]^ Histopathological examination combined with immunohistochemistry (IHC) remains the most widely used approach for diagnosing MFS, allowing differentiation from other spindle cell neoplasms.^[7]^ The first description of the pathological characteristics of MFS was by Vasudev and Harris in 1978.^[8]^ No highly invasive thyroid MFS cases have been reported in China. Unlike conventional malignant thyroid tumors, which originate from the epithelial cells of thyroid follicles, MFS arises from the interstitial (mesenchymal) tissue. These tumors typically comprise spindle-shaped cells and characteristically express markers such as vimentin and smooth muscle actin features that distinguish them from epithelial-derived neoplasms. MFS is significantly aggressive, with a strong tendency to infiltrate surrounding tissues and vascular structures, often leading to early distant metastasis. This report presents a rare case of highly invasive thyroid MFS in an adult, highlighting its immunohistochemical and pathological features, diagnostic process, and clinical management. This study had approved by the Ethics Committee of Liangzhou Hospital in Wuwei (lyll20240104), the patient and her next of kin gave their informed consent.

## 2. Case report

### 2.1. Clinical data

The patient was a 61-year-old woman with a history of a thyroid mass spanning over 30 years. On February 20, 2019, magnetic resonance imaging confirmed the presence of a malignant thyroid tumor, and surgical intervention was advised. She also had a 7-year history of hypertension. However, due to poor blood pressure control and elevated surgical risk, the procedure was not performed. Over the past month, the thyroid mass had enlarged rapidly. The patient began experiencing hoarseness and shortness of breath, prompting readmission to our hospital.

**Figure 1.**
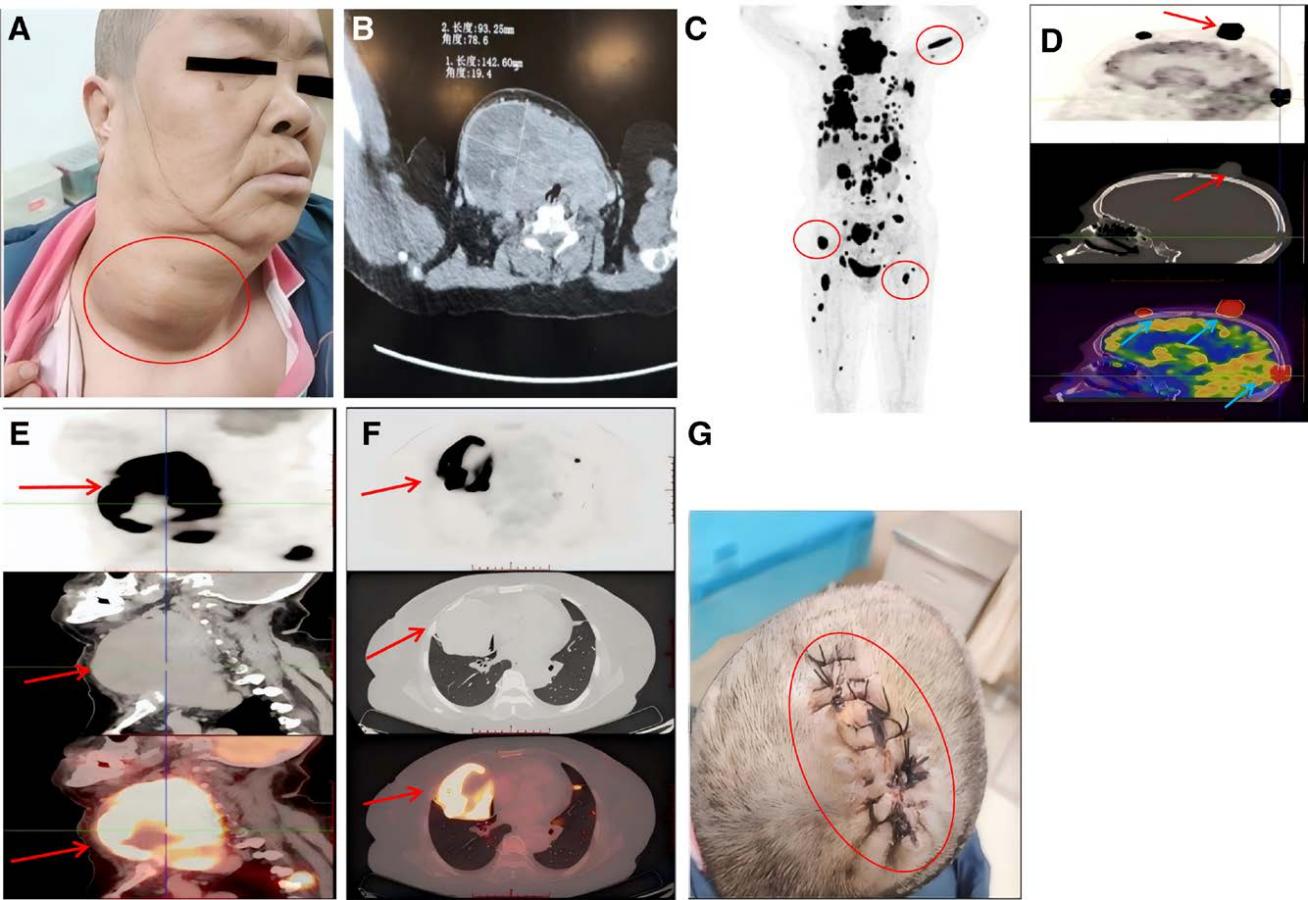
(A) Thyroid lesion. (B) CT measurement of thyroid lesions. (C) Whole-body bone scintigraphy multiple bone metastases. (D) PET CT imaging of scalp metastases. (E) PET-CT imaging of thyroid lesions. (F) PET CT imaging of lung metastatic lesions. (G) After scalp mass excision. CT = computed tomography, PET-CT = positron emission tomography-computed tomography.

On physical examination, multiple nodular lesions were observed on the scalp. The neck appeared asymmetrical but showed no resistance on palpation. The trachea was deviated to the left. Thyroid morphology was abnormal. A large solid mass was palpable in the anterior neck (Fig. 1A), measuring approximately 14.26 × 9.325 cm (Fig. 1B). The mass had poor mobility and slight tenderness but no associated tremor. Given the long-standing nature of the thyroid mass and its recent rapid growth, the possibility of undifferentiated carcinoma could not be excluded. A thyroid biopsy was therefore performed on January 5, 2024, for definitive diagnosis.

Routine blood tests and tumor marker analysis revealed no significant abnormalities. An electronic laryngoscopy showed right vocal cord paralysis. Computed tomography (CT) scans of the cervical, thoracic, and abdominal regions (plain and contrast-enhanced) identified a space-occupying lesion in the thyroid. Multiple pulmonary nodules and a soft tissue mass in the fourth posterior rib on the right side were detected. Further findings included hepatic nodules in the left lobe and masses in the pancreas and spleen, which suggest metastatic disease. Due to suspected metastases in the lungs and right rib, a biopsy of the right pulmonary lesion was performed. Emission CT (Fig. 1C) showed multiple skeletal sites with abnormal metabolic activity, consistent with bone metastases.

The patient had previously undergone a hysterectomy and bilateral adnexectomy for an ovarian cystic tumor at another hospital on August 30, 2014.^[9]^ Postoperative pathology indicated a borderline ovarian tumor. She did not receive adjuvant therapy but was followed up regularly. To further evaluate the extent of metastasis and determine tumor staging, a positron emission tomography-CT scan was performed on January 10, 2024 (Fig. 1D–F). Imaging revealed slightly hypodense or isodense nodules and masses located in the thyroid, left frontal lobe and subdural region, both lungs, liver, spleen, peripancreatic area and pancreatic tail, left kidney, bilateral cervical regions, mesentery of the left abdomen, perivascular areas of the right iliac bone, multiple bones (including the skull), subcutaneous tissues and muscles of the bilateral scalp, left lower lip and torso, and at the rectosigmoid junction. Based on clinical history and imaging, the largest lesion was located in the thyroid, with evidence of tracheal compression. The multiple lesions in both lungs, bone, and subcutaneous tissues were morphologically consistent with metastatic disease. Although the liver, pancreas, and spleen had fewer lesions, the radiographic appearance did not support a primary origin in those organs.

These findings suggested that the primary tumor was most likely a malignant thyroid neoplasm, although differential diagnoses such as lymphoid or neuroendocrine tumors could not be excluded. A pathological biopsy was recommended for confirmation. During hospitalization, the scalp mass began to bleed. On January 13, 2024, the patient underwent lumpectomy of the scalp lesion (Fig. 1G), and the excised tissue was submitted for pathological analysis.

## 3. Pathological and IHC examinations

### 3.1. Thyroid mass

**Figure 2.**
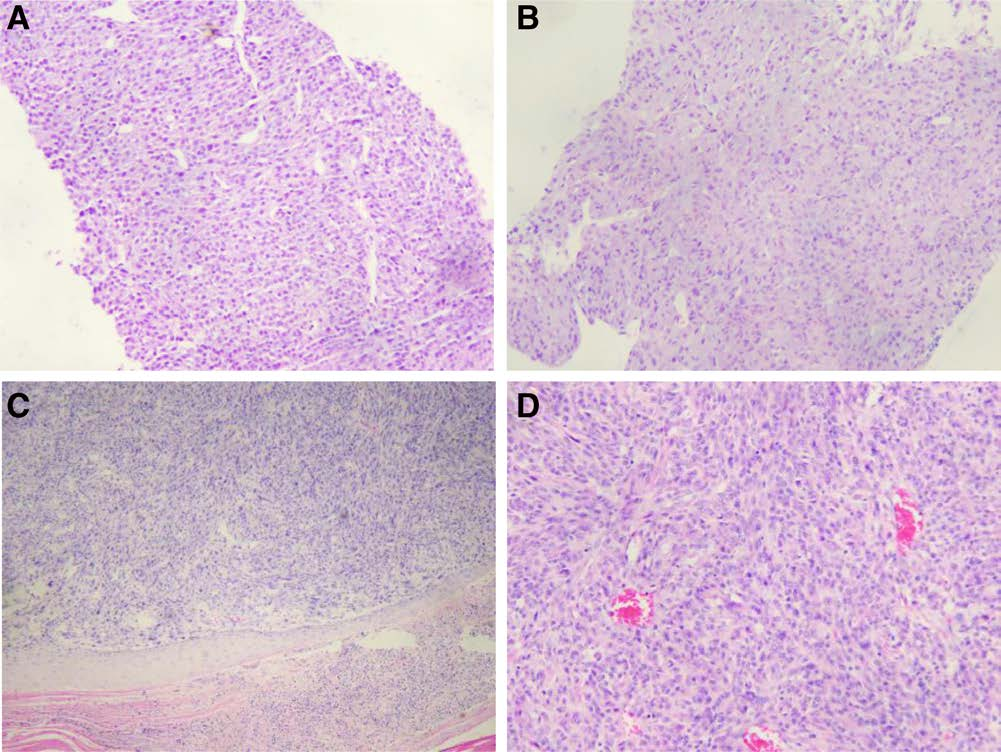
Pathological each tissue. (A) Thyroid puncture, hematoxylin–eosin stain (HE). Original magnification (HE × 100). (B) Right lung puncture. Hematoxylin–eosin stain (HE), original magnification (HE × 100). (C) Scalp mass, hematoxylin–eosin stain (HE). Original magnification (HE × 40). (D) Scalp mass. Hematoxylin–eosin stain (HE), original magnification (HE × 100).

**Figure 3.**
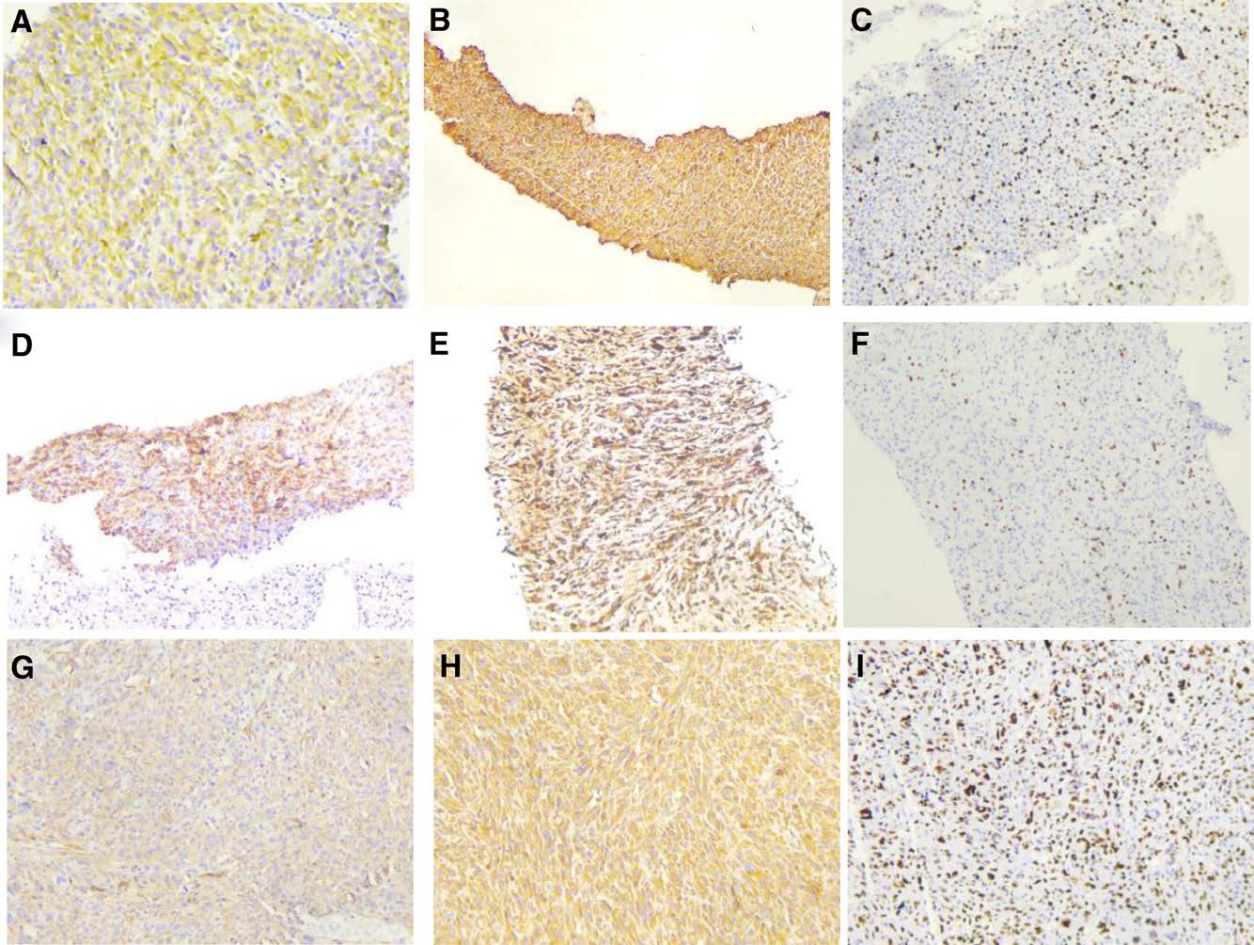
Tumor cell immunohistochemistry (IHC). (A) Positive expression of SMA in thyroid puncture tissue (IHC × 100). (B) Positive expression of vimentin in thyroid puncture tissue (IHC × 100). (C) Positive expression of Ki-67 in thyroid puncture tissue (20%) (IHC × 100). (D) Positive expression of SMA in right lung puncture (IHC × 100). (E) Positive expression of vimentin in right lung puncture (IHC × 100). (f) Positive expression of Ki-67 in right lung puncture (IHC × 100). (G) Positive expression of SMA in scalp mass (IHC × 100). (H) Positive expression of vimentin in scalp mass (IHC × 100). (I) Positive expression of Ki-67 in scalp mass (80%) (IHC × 100). IHC = immunohistochemistry, SMA = smooth muscle actin.

Biopsy of the thyroid mass revealed a disorganized proliferation of short spindle-shaped or epithelioid cells, some showing eosinophilic cytoplasm and focal areas of necrosis. Various mitotic figures were observed, with a mitotic index of 10 per 10 high-power fields (Fig. 2A). IHC staining (Fig. 3A–C) demonstrated the following expression profile:

*Positive markers*: vimentin (+), smooth muscle actin (SMA, +), Ki-67 (20%+), cyclin D1 (+), and galectin-3 (focally +).

*Negative markers*: S-100, CD34, myogenin, HMB45, CD99, CD31, PAX-8, thyroglobulin, synaptophysin, P63, P53 (wild-type?), thyroid transcription factor-1, Bcl-2, calcitonin, mast cell tryptase, cytokeratin 19, CD56, thyroid peroxidase, E-cadherin, cytokeratin pan (Ckpan), and epithelial membrane antigen were all negative.

### 3.2. Right lung masses

Histopathologically examining the right lung mass biopsy revealed a disorganized proliferation of hyperplastic short spindle-shaped or epithelioid cells. Some of these cells displayed eosinophilic cytoplasm and focal necrosis. Mitotic figures were observed at a rate of 8 per 10 high-power fields (Fig. 2B). Based on the histomorphological features and IHC profile, the findings were consistent with those observed in the thyroid mass biopsy.

IHC staining (Fig. 3D–F) demonstrated the following profile:

*Positive markers*: vimentin (+), SMA (+), and Ki-67 (10%+).

*Negative markers*: desmin, CD117, estrogen receptor, P16, caldesmon, and CD45 (positive only in lymphocytes).

### 3.3. Scalp masses

Histopathological analysis of the scalp mass biopsy revealed a proliferation of spindle-shaped cells arranged in irregular or fascicular patterns. The nuclei varied in size, with some appearing large and atypical, showing coarse chromatin. Frequent mitotic figures were observed, with a mitotic index of 13 per 10 high-power fields. Residual tumor tissue was noted at the basal margin of the specimen (Fig. 2C and D).

IHC staining (Fig. 3G–I) demonstrated the following results:

*Positive markers*: SMA (+), vimentin (diffuse +), and Ki-67 (80%+).

### Negative

*markers*: calponin, S-100, CD34, desmin, CD99, CD68, and pan-cytokeratin (CKpan, negative in tumor cells). P53 showed a wild-type expression pattern.

## 4. Outcome and treatment

### 4.1. Outcome

The immunophenotype of MFS closely resembles that of myofibroblasts, which originate from fibroblasts, express myogenic markers, and possess a cytoskeleton composed primarily of vimentin.^[10]^ Vimentin expression is associated with at least one myogenic biomarker, SMA^[11,12]^; thus, MFS is usually strongly positive for vimentin and SMA/α-SMA. Immunohistochemical analysis of this patient’s thyroid, lung, and scalp lesions showed positive staining for both vimentin and SMA, consistent with previously reported findings in MFS cases. Ki-67, a well-established marker of tumor cell proliferation, plays a critical role in assessing tumor aggressiveness; however, its diagnostic threshold varies depending on tumor type, histological subtype, and clinical purpose. For example, defined cutoffs (≤3%, 3–20%, >20%) are commonly applied in the World Health Organization classification of neuroendocrine tumors (G1/G2/G3) to guide therapeutic decision-making.^[13,14]^ Threshold values (e.g., ≤14–20% vs >14–20%) are often used alongside hormone receptor status to distinguish between luminal A-like and luminal B-like subtypes.^[15,16]^ However, the use of Ki-67 is limited by issues related to interobserver variability, intratumoral heterogeneity, and a lack of standardized assessment protocols,^[17,18]^ and Ki-67 shall be interpreted based on the specific tumor type, other pathological features, latest guidelines, and lab standards.^[19]^

In this case, the Ki-67 proliferation index was 80%, indicating a highly aggressive tumor with rapid growth and poor prognosis. Immunohistochemical staining revealed the absence of thyroglobulin, thyroid transcription factor-1, and PAX-8 expression, effectively excluding malignancies originating from thyroid follicular epithelial cells. The lack of cCT expression ruled out tumors derived from thyroid parafollicular (C) cells. Similarly, the absence of cytokeratin 19 and CKpan expression excluded epithelial-derived malignancies. Negative mast cell tryptase, S-100, and HMB45 staining suggested that schwannoma and melanoma were unlikely. The absence of myogenin expression ruled out rhabdomyosarcoma, and negative CD34 staining excluded solitary fibrous tumors. The lack of CKpan expression also argued against carcinosarcoma or spindle cell carcinoma.

To further investigate the genetic profile, high-throughput sequencing was performed, targeting the exonic regions of 437 genes, including relevant introns, alternative splicing sites, and microsatellite regions. No ALK gene mutations were detected, excluding the possibility of an inflammatory myofibroblastic tumor. Based on the morphological characteristics observed through both hematoxylin–eosin and IHC staining, the thyroid, right lung, and scalp masses were identified as mesenchymal-derived spindle cell tumors, consistent with tumors of myofibroblastic origin. The possibility of metastasis from the previously diagnosed borderline ovarian tumor was excluded. Taking into account the patient’s clinical history, presenting symptoms and signs, auxiliary diagnostic findings, and applying the French National Cancer Council^[20,21]^ grading system for soft tissue sarcomas (adopted by the National Cancer Institute), the diagnosis was confirmed as a high-grade, highly invasive MFS originating in the thyroid. The tumor was classified as grade 3 according to the French National Cancer Council criteria. A detailed breakdown of the histological grading for the thyroid, right lung, and scalp lesions is presented in Table 1.

**Table 1:** Standards of histological levels of FNCLCC and the histological scores of the patient.

Histological parameters	Standards of histological levels of FNCLCC	Thyroid mass	Right lung masses	Scalp masses
*Tumor differentiation*				
Score 1	Sarcoma similar to normal mesenchymal tissues in adults (e.g., hyperdifferentiated liposarcoma)	Unclear sarcoma	Undifferentiated sarcoma	Unclear sarcoma
Score 2	Sarcoma that can be histologically typed (e.g., mucinous liposarcoma)			
Score 3	Embryonal or undifferentiated sarcoma, unclear sarcoma			
*Karyokinesis count*				
Score 1	0–9/10HPF	Karyokinesis count = 10/10HPF	Karyokinesis count = 8/10HPF	Karyokinesis count = 13/10HPF
Score 2	10–19/10HPF			
Score 3	≥20/10HPF			
*Tumors necrosis*				
Score 0	N.A.	Tumor necrosis area > 50%	Tumor necrosis area > 50%	Tumor necrosis area < 50%
Score 1	<50%			
Score 2	≥50%			
*Histological levels*				
Level 1	Overall score = 2, 3	Overall histological score of tumor = 7	Overall histological score of tumor = 6	Overall histological score of tumor = 6
Level 2	Overall score = 4, 5			
Level 3	Overall score = 6, 7, 8			

FNCLCC = French National Cancer Council.

### 4.2. Clinical pre-targeted therapy

Given the presence of multiple secondary malignant lesions (in the scalp, lungs, bones, liver, spleen, and pancreas), the tumor’s high degree of malignancy, widespread metastasis, and invasion of the recurrent laryngeal nerve, which rendered complete surgical resection unfeasible, the patient was deemed unsuitable for surgery. To explore potential options for targeted therapy, genetic testing was conducted using high-throughput sequencing on peripheral blood and thyroid and lung biopsy specimens at Nanjing Shihe Medical Laboratory. The sequencing panel covered approximately 1.53 Mb of genomic regions, including exons, fusion-related introns, alternatively spliced regions, and microsatellite instability-specific domains across 437 cancer-related genes. The analysis identified point mutations, small insertion-deletion variants, gene fusions, copy number alterations, and microsatellite status and tumor mutational burden assessment. Sequencing was performed regarding the GRCh37/hg19 human genome. As shown in Table 2, genetic analysis identified a missense mutation in exon 3 of the *NRAS* gene; in thyroid tumors, this mutation is associated with sensitivity (grade C) to the targeted binimetinib.^[22]^ Further mutations were detected in exons 5 and 8 of the *TP53* gene, for which anlotinib was recommended as an oral treatment option.^[23]^ A missense mutation was also found in exon 14 of the *PDGFRB* gene, which plays a role in metabolic immune suppression within the tumor microenvironment (TME). This suggests that the patient would derive limited benefit from immunotherapy.^[24]^ Due to the aggressive progression of the disease, the patient and her family declined further treatment. She passed away on January 31, 2024, likely as a result of widespread distant metastases.

**Table 2:** Tumor-specific mutations.

Gene	Mutation	Mutant type	Plasma abundance	Tissue abundance
NRAS	Missense mutation of exon 3 in p.Q61R	c.182A>G(p.Q61R)	7.86%	39.99%
TP53	Missense mutation of exon 5 in p.F134L	c.400T>C(p.F134L)	4.22%	
	Non-frameshift deletion mutation of Exon 8 in p.L265del	c.792_794del(p.L265del)	0.05%	41.61%
EIF1AX	Shear mutation of intron 5 in c.338-1G>A	c.338-1G>A	6.53%	38.58%
PDGFRB	Missense mutation of exon 14 in p.N666K	c.1998C>A(p.N666K)	3.49%	40.51%
	Missense mutation of exon 12 In P.P560R	c.1679C>G(p.P560R)	6.72%	36.08%
TERT	Missense mutation of exon 14 in p.N666K	C.-124C>T	5.63%	36.08%

## 5. Discussion

Gabbiani and Majno^[25]^ described myofibroblasts as cells showing both morphological and functional features of fibroblasts and smooth muscle cells. These cells are typically observed in tissues undergoing repair after injury and benign tumors with local invasive behavior. The MFS represents the malignant counterpart of these cells. Vasudev and Harris reported the first documented MFS case in 1978.^[8]^ The most common presentation of MFS is a painless, progressively enlarging mass. The tumor often arises in the head and neck region, including the retroauricular area, face, tonsils, thyroid gland, neck, and scalp, as well as in the trunk and extremities. According to the National Cancer Institute System grading criteria for soft tissue tumors,^[20,21]^ MFS is classified into low-, intermediate-, and high-grade categories. Low-grade MFS typically shows local invasive growth with a tendency to recur. Intermediate-grade MFS shows higher recurrence rates and is prone to distant metastasis. High-grade MFS is characterized by marked cellular polymorphism,^[26,27]^ poor prognosis, and high mortality.

### 5.1. Clinical features

Thyroid sarcomas can originate from various histological subtypes, including fibrosarcoma, mixed osteosarcoma, leiomyosarcoma, vascular sarcoma, and carcinosarcoma.^[28–32]^ Morphologically, they resemble sarcomas found in other tissues and organs. Most patients have a prolonged history of thyroid enlargement before diagnosis. These tumors typically show rapid progression, a firm consistency, ill-defined borders, and strong adherence to surrounding structures. As a result, the mass often displays poor mobility during swallowing. Thyroid sarcomas may involve one or both lobes and frequently invade adjacent anatomical structures such as the recurrent laryngeal nerve, leading to vocal cord paralysis and hoarseness. Invasion of the trachea and esophagus can further result in respiratory and swallowing difficulties. Hematogenous spread often occurs early in the disease course, whereas lymph node metastasis is uncommon. Although surgery remains the mainstay of treatment, recurrence following resection is common due to the aggressive nature of these tumors.

Reports on MFS^[33–35]^ indicate a wide anatomical distribution, with frequent involvement of the head and neck region. MFS typically presents as a painless, enlarging mass. In cases involving the nasal cavity, symptoms may include nasal obstruction, epistaxis, and rhinorrhea, whereas orbital tumors are often associated with proptosis. When MFS arises in bone, radiographs usually reveal destructive, osteolytic lesions without periosteal reaction. Low-grade MFS tends to follow a more indolent clinical course and is primarily managed with surgical excision; recurrence is rare following complete resection, and outcomes are generally favorable. Intermediate-grade MFS is associated with higher recurrence rates and frequent distant metastases. High-grade MFS demonstrates an aggressive clinical course, poor prognosis, and elevated short-term mortality.

No cases of primary myofibroblastic sarcoma of the thyroid have been documented. Most MFSs in bone and soft tissue are classified as low-grade myofibroblastic sarcomas. High-grade MFS, due to its histopathological resemblance to fibrosarcoma, undifferentiated/unclassified soft tissue sarcoma, or undifferentiated pleomorphic sarcoma, is sometimes grouped under these entities or labeled as malignant fibrous histiocytoma, reflecting ongoing classification challenges.

The present case represents a rare and highly malignant form of thyroid MFS. The patient had a long-standing history of thyroid disease, with the current tumor showing rapid growth during the month before admission. The mass was firm, poorly demarcated, and had invaded the recurrent laryngeal nerve, resulting in vocal cord paralysis and hoarseness. Widespread hematogenous metastases involving the scalp, lungs, bones, and other sites had also developed. A definitive diagnosis was made through core needle biopsy of both the primary and metastatic lesions, supplemented by surgical biopsy and expert pathological consultation. Due to the tumor’s large size, high-grade malignancy, and extensive metastatic spread, complete surgical excision was deemed unfeasible and unlikely to confer clinical benefit.

Genetic testing was conducted on tumor tissue to explore targeted therapeutic options. Based on the findings, treatment with anlotinib was recommended. Unfortunately, due to the rapid progression of the disease, the patient succumbed before treatment could be initiated.

### 5.2. Treatment and prognosis

Low-grade myofibroblastic sarcoma in the head and neck region has been reported to show invasive behavior and a high propensity for local recurrence. Cases with intermediate malignancy can progress to distant metastasis and may result in mortality. Patients with highly malignant MFS typically have poor prognoses and elevated mortality rates. For tumors detected at an early stage without extensive metastasis, comprehensive treatment involving surgery, radiotherapy, and chemotherapy is recommended. Patients with widespread tumor infiltration or distant metastases, for whom surgery is not feasible, should be considered for chemoradiotherapy, genetic profiling for potential targeted therapies, or immunotherapy.

MFS is a rare mesenchymal tumor whose molecular features are not yet fully understood. Research into myofibroblast differentiation has revealed that MFS is marked by abnormal activation of the transforming growth factor-β /SMAD signaling pathway, which drives the transformation of fibroblasts into myofibroblasts. This pathway also contributes to an immunosuppressive TME by impairing CD8^+^ T cell function and promoting the differentiation of regulatory T cells.^[36]^ The TME plays a pivotal role in the progression and therapeutic resistance of thyroid MFS. Its immunosuppressive nature is largely driven by tumor-associated macrophages, cancer-associated fibroblasts (CAFs), and regulatory T cells.

A recent single-cell study of non-small cell lung cancer demonstrated that SELENOP-expressing macrophages interact with antigen-presenting CAFs at tumor boundaries to form inhibitory cellular hubs. These structures facilitate immune evasion and create physical barriers to immune cell infiltration.^[37]^ In addition to cellular interactions, persistent activation of the MAPK/ERK signaling cascade leads to overamplification of receptor tyrosine kinases such as PDGFRβ and FGFR2, promoting metabolic immune suppression within the TME.^[24]^

Furthermore, Liu et al^[38]^ reported that overexpression of tryptophan 2,3-dioxygenase (TDO2) in myofibroblasts at pulmonary metastatic sites of breast cancer may also be relevant in thyroid MFS. TDO2, a rate-limiting enzyme in tryptophan metabolism, produces kynurenine, suppressing T cell function and protecting tumor cells from ferroptosis, thus contributing to immune escape.

These findings offer valuable insights for developing targeted therapies in thyroid MFS. Epigenetic dysregulation also plays a role in tumor progression. For example, downregulation of the miR-200 family removes repression of ZEB1/ZEB2, thereby promoting EMT.^[24]^ Furthermore, ECM components secreted by CAFs can form dense physical barriers that hinder T cell infiltration and impede drug delivery, further contributing to immune resistance.^[37]^

Zhang et al^[39]^ found that expression of MFSD2A positively correlates with the efficacy of anti-PD-1 therapy in gastric cancer. MFSD2A enhances immune response by reducing TGF-β1 secretion, restoring TME homeostasis, and promoting CD8^+^ T cell activation via inhibition of COX2-prostaglandin synthesis. These findings suggest combining COX2 inhibitors (e.g., celecoxib) with immune checkpoint inhibitors may offer a promising therapeutic strategy for thyroid MFS.

In conclusion, the clinical diagnosis of highly malignant thyroid MFS remains challenging, particularly in the early stages, due to its rarity and nonspecific presentation. The disease typically shows aggressive progression and is associated with a poor short-term survival rate. Clinicians should maintain a high index of suspicion in patients with a longstanding history of thyroid disease, especially when there is rapid enlargement of the mass or recent onset of local compressive symptoms.

The limitations of this case report: the case report describes a highly aggressive thyroid myofibroblastic sarcoma, which is only a single case report. The disease progressed rapidly, and the patient died within a short period, with no treatment outcomes available. The diagnosis was solely based on pathological results from core needle biopsies of the thyroid and right lung metastatic lesions, as well as resection of the scalp metastatic lesion. Additionally, the immunohistochemical staining for calponin in the scalp metastatic lesion was negative, posing diagnostic challenges.

One of the primary obstacles in advancing the understanding and management of thyroid MFS is the limited availability of clinical samples. Future progress in precision therapy is likely to rely on the development of combination immunotherapies such as CD47/PD-1 dual blockade and TGF-β trap proteins 36 alongside strategies aimed at reprogramming the tumor microenvironment, including metabolic interventions targeting TDO2 and IDO1 24. Moreover, efforts should focus on establishing subtype-specific targeted therapies and leveraging artificial intelligence-based predictive models to enable individualized treatment approaches.^[40]^

## Author contributions

**Formal analysis:** Jianwei Zhang.

**Investigation:** Liuxue Ma, Xin Li.

**Methodology:** Liuxue Ma, Huijuan Wang.

**Project administration:** Liuxue Ma.

**Resources:** Zixin Dai.

**Writing – review & editing:** Liuxue Ma, Huijuan Wang, Jinxia Gao.
